# Human IL-17 and TNF-α Additively or Synergistically Regulate the Expression of Proinflammatory Genes, Coagulation-Related Genes, and Tight Junction Genes in Porcine Aortic Endothelial Cells

**DOI:** 10.3389/fimmu.2022.857311

**Published:** 2022-06-30

**Authors:** Weilong Li, Pengfei Chen, Yanli Zhao, Mengtao Cao, Wenjun Hu, Litao Pan, Huimin Sun, Dongsheng Huang, Hanxi Wu, Zhuoheng Song, Huanli Zhong, Lisha Mou, Shaodong Luan, Xiehui Chen, Hanchao Gao

**Affiliations:** ^1^ Department of Nephrology, Shenzhen Longhua District Central Hospital, Affiliated Central Hospital of Shenzhen Longhua District, Guangdong Medical University, Shenzhen, China; ^2^ Department of Anesthesiology, The 305 Hospital of People's Liberation Army of China (PLA), Beijing, China; ^3^ Department of Acupuncture and Massage, Shenzhen Second People’s Hospital, The First Affiliated Hospital of Shenzhen University, Shenzhen, China; ^4^ Department of Medical Administration, People’s Hospital of Shenzhen Longhua Branch, Shenzhen, China

**Keywords:** xenotransplantation, immune rejection, cytokines, IL-17, TNF-α, PAECs, inflammation, coagulation

## Abstract

Immune rejection is the major limitation for porcine xenograft survival in primate recipients. Proinflammatory cytokines play important roles in immune rejection and have been found to mediate the pathological effects in various clinical and experimental transplantation trials. IL-17 and TNF-α play critical pathological roles in immune disorders, such as psoriasis and rheumatoid arthritis. However, the pathological roles of human IL-17 (hIL-17) and human TNF-α (hTNF-α) in xenotransplantation remain unclear. Here we found that hIL-17 and hTNF-α additively or synergistically regulate the expression of 697 genes in porcine aortic endothelial cells (PAECs). Overall, 415 genes were found to be synergistically regulated, while 282 genes were found to be additively regulated. Among these, 315 genes were upregulated and 382 genes were downregulated in PAECs. Furthermore, we found that hIL-17 and hTNF-α additively or synergistically induced the expression of various proinflammatory cytokines and chemokines (*e*.*g*., IL1α, IL6, and CXCL8) and decreased the expression of certain anti-inflammatory genes (*e*.*g*., IL10). Moreover, hIL-17 plus hTNF-α increased the expression of IL1R1 and IL6ST, receptors for IL1 and IL6, respectively, and decreased anti-inflammatory gene receptor expression (IL10R). hIL-17 and hTNF-α synergistically or additively induced CXCL8 and CCL2 expression and consequently promoted primary human neutrophil and human leukemia monocytic cell migration, respectively. In addition, hIL-17 and hTNF-α induced pro-coagulation gene (SERPINB2 and F3) expression and decreased anti-coagulation gene (TFPI, THBS1, and THBD) expression. Additionally, hIL-17 and hTNF-α synergistically decreased occludin expression and consequently promoted human antibody-mediated complement-dependent cytotoxicity. Interestingly, hTNF-α increased swine leukocyte antigen (SLA) class I expression; however, hIL-17 decreased TNF-α-mediated SLA-I upregulation. We concluded that hIL-17 and hTNF-α likely promote the inflammatory response, coagulation cascade, and xenoantibody-mediated cell injury. Thus, blockade of hIL-17 and hTNF-α together might be beneficial for xenograft survival in recipients.

## Introduction

Organ transplantation is an effective way of end-stage organ failure therapy. However, the shortage of human donors is a major limitation that prevents clinical application. Xenotransplantation is a promising way to solve this problem (1). Pigs are considered to be the most suitable organ donor animal (2, 3), and gene-modified pigs lead to increased xenograft survival; however, immune rejection is still a major hurdle in the survival of xenografts in primate recipients (4–10).

In pig-to-human organ transplantation, porcine vascular endothelial cells (ECs), which are the first cells to interact with the human immune system, play a critical role in the immune rejection of xenografts (11). Porcine ECs are activated by human-derived cytokines or chemokines and attacked by the human immune system in pig-to-human xenotransplantation (12). EC injury and dysfunction are critical for the inflammation and coagulation response, which will decrease pig organ survival in human recipients (13).

Cytokines play critical roles in inflammatory responses. In xenotransplantation, many proinflammatory cytokines are produced, including IL-17 and TNF-α (14). We previously found that human IL-6, IL-17, IL-1β, and TNF-α obviously activated porcine ECs, whereas human IFN-γ did not activate porcine ECs (12). Human TNF-α largely activates the NF-κB and mitogen-activated protein kinase (MAPK) pathways and induces downstream proinflammatory and procoagulation gene expression in porcine aortic endothelial cells (PAECs) (12). Human IL-17 also activates the NF-κB and MAPK pathways in PAECs (12). Several studies have reported that IL-17 and TNF-α additively or synergistically induce the expression of many genes to promote the development of various diseases, such as psoriasis and rheumatoid arthritis (15, 16). We also found that hIL-17 and hTNF-α additively or synergistically induce E-selectin, ICAM-1, IL-6, CXCL8, and CCL2 expression in PAECs (12). However, whether human IL-17 and TNF-α additively or synergistically induce the expression of certain genes to promote immune rejection in xenotransplantation remains unclear.

To answer this question, we stimulated PAECs with hIL-17, hTNF-α, or hIL-17 plus hTNF-α *in vitro*. We checked the mRNA levels in hIL-17- or hTNF-α-treated PAECs *via* transcriptome sequencing and analyzed the data using bioinformatics tools. We found that hIL-17 and hTNF-α additively or synergistically regulate the expression of hundreds of genes in PAECs. Many cytokines and chemokines (and some receptors for these genes) are regulated by IL-17 plus TNF-α. IL-17 plus TNF-α synergistically and additively induced CXCL8 and CCL2 expression and consequently promoted human neutrophil and THP-1 cell migration, respectively. Moreover, hIL-17 and hTNF-α additively or synergistically induced procoagulation gene (SERPINB2 and F3) expression and decreased anti-coagulation gene (TFPI, THBS1, and THBD) expression. Human IL-17 and hTNF-α also decreased occludin expression and consequently promoted human antibody-mediated complement-dependent cell injury. Here we demonstrate that hIL-17 and hTNF-α likely promote xenograft rejection in xenotransplantation.

## Materials and Methods

### Reagents

Recombinant human IL-17, recombinant human TNFα, and recombinant porcine IFN-γ were purchased from R&D Systems (Minneapolis, MN, USA). Anti-actin antibody was purchased from Cell Signaling Technology (Boston, MA, USA), anti-occludin antibody was obtained from Thermo Fisher Scientific (Rockford, IL, USA), anti-FITC-labeled SLA class I antibody (SLA-I) was obtained from Bio-Rad (Hercules, CA, USA), and Cell Counting Kit-8 (CCK8) was purchased from Dojindo Laboratories (Kumamoto, Japan). The CCR2 (CCL2 receptor)-specific inhibitor RS504393 was from MedChemExpress (Shanghai, China).

### Preparation of Porcine Aortic Endothelial Cells

PAECs were isolated from wild-type or *GGTA1/CMAH* double-knockout (DKO) Chinese domestic miniature Wuzhishan pig arteries as previously described (17). In brief, freshly harvested porcine arteries were treated with 0.05% collagenase B (Roche Applied Science, Indianapolis, IN, USA). We collected the cells and washed them with washing medium [RPMI 1640 containing 10% heat-inactivated fetal bovine serum (FBS)]. The isolated PAECs were cultured with endothelial cell medium containing 10% (vol/vol) FBS (cat. no. 0025), 1% (vol/vol) penicillin/streptomycin solution (P/S, cat. no. 0503), and 1% (vol/vol) endothelial cell growth supplement (ECGS, cat. no. 1052) at 37°C with 5% CO_2._ The culture medium was purchased from Sciencell (San Diego, CA, USA).

### Western Blotting

PAECs were harvested after washing with ice-cold phosphate-buffered saline (PBS), lysed for 30 min in ice-cold RIPA lysis buffer supplemented with 10 mM NaF, 1 mM Na_3_VO_4_, 1 mM phenylmethylsulfonyl fluoride, and protease inhibitor cocktail (Roche, Indianapolis, IN, USA), and separated *via* 10% SDS–PAGE. After transfer onto polyvinylidene fluoride (PVDF) membranes (Millipore, Billerica, MA, USA), the proteins on the PVDF membranes were blocked using 5% non-fat dried milk dissolved in TBST (20 mM Tris-HCl, 150 mM NaCl, pH 7.6) buffer supplemented with 0.1% (vol/vol) Tween 20 at room temperature for 1 h. After washing, the PVDF membranes were incubated with primary antibody overnight at 4°C and then washed with TBST. After incubation with secondary antibody for 1 h at room temperature, the blots were visualized with enhanced chemiluminescence detection reagents (Millipore).

### Real-Time PCR

The procedure for real-time PCR has been reported previously (18). Briefly, total RNA was extracted from cells or tissues with TRIzol^®^ Reagent (Invitrogen, Shanghai, China). cDNA samples were synthesized with PrimeScript™ RT Master Mix (Takara Bio, Dalian, LN, China). The levels of the genes of interest were quantified using TB Green^®^ *Premix Ex Taq* ^™^ (Tli RNaseH Plus) (Takara Bio). The expression level of the genes was calculated using the 2^-ΔΔCt^ method and normalized to the rpl13a expression level. cDNA amplification was performed using a ViiA 7 Real-Time PCR system (Applied Biosystems, Foster City, CA, USA), and the oligonucleotide primer sequences are shown in **Supplementary Table S1**.

### Flow Cytometry Analysis

SLA-I binding was assessed using flow cytometry as previously reported (12). Porcine aortic endothelial cells were collected and washed once with PBS, and 1 × 10^6^ cells were resuspended in 100 μl PBS containing 1% BSA and then stained with FITC-labeled SLA-I antibody. Isotype-matched antibody was used as a negative control. After incubation for 30 min at 4°C in the dark, the cells were washed once with PBS, resuspended in 100 μl PBS containing 1% BSA and detected with a BD FACS Aria II flow cytometer (Franklin Lakes, NJ, USA). The extent of SLA-I binding to PAECs was evaluated by geometric mean fluorescence intensity (Gmean).

### Human Antibody-Mediated Complement-Dependent Cytotoxicity

The protocol for human antibody-mediated complement-dependent cytotoxicity assessment has been previously reported (11). In brief, PAECs (4 × 10^3^) were seeded into 96-well plates and treated with medium (as a negative control), hIL-17 (100 ng/ml), hTNF-α (2 ng/ml), or hIL-17 plus hTNF-α for 48 h. The supernatant was removed and replaced with RPMI-1640 medium containing 20% pooled human serum [experimental group—the serum was pooled from several healthy volunteers (*n* = 20), including all ABO blood types] or 20% heat-inactivated human serum (control group) for 2 h. After 2 h, CCK8 assays were used to assess the viability of PAECs. The supernatant was removed and replaced with RPMI-1640 medium containing 10% CCK8 reagent for 2 h. At 2 h later, the absorbance values of the wells at OD450 were measured using a multiscan GO spectrophotometer (Thermo Fisher, Vantaa, Finland). The percentage of cell death (cytotoxicity) was calculated according to the following formula:


% cytotoxicity= (OD of control group − OD of experimental group)/OD of control group × 100

### Transwell Assay

The chemotaxis assay procedure has been reported previously (19). In brief, isolation of human neutrophils using a human neutrophil isolation kit was performed according to the manufacturer’s instructions. The chemotaxis assay was performed in 24-well plates using 6.5 μm (for THP‐1) or 3 μm (for human neutrophils) transwell inserts with 5-mm pore polycarbonate membranes (Corning). The PAECs were treated with hIL-17 (100 ng/ml), hTNF-α (2 ng/ml), or hIL-17 plus hTNF-α for 48 h, the supernatant was collected and then added to the lower transwell chamber, and 25 × 10^4^ THP-1 or human neutrophils were seeded in the upper chamber. The transmigration assay was performed for 2 h at 37°C. For CCR2 inhibitor assay, THP-1 cells were treated with 10 μM RS504393 for 1 h and then seeded in the upper chamber. The membranes containing the migrated cells were carefully excised. Images were obtained with a microscope, and the migrated cells were counted. The migration data are presented as the number of migrating cells/field.

### Transcriptome Sequencing

PAECs were treated with rhIL-17 (100 ng/ml), rhTNF-α (2 ng/ml), or rhIL-17 plus rhTNF-α for 0 or 6 h. RNA was extracted from the treated PAECs using TRIzol reagent (Invitrogen) and was utilized to construct the final library. The sequencing library was generated using the VAHTS mRNA-seq v2 Library Prep Kit for Illumina^®^ (Vazyme, NR601, Nanjing, JS, China) following the manufacturer’s recommendation. Library concentration was measured using a Qubit^®^ RNA Assay Kit and Qubit^®^ 3.0 for preliminary quantification. Insert size was assessed using an Agilent Bioanalyzer 2100 system, and after the insert size was consistent with expectations, the qualified insert size was accurately quantified *via* qPCR using a Step One Plus Real-Time PCR system (Applied Biosystems). Clustering of the index-coded samples was performed on a cBot Cluster Generation System (Illumina, San Diego, CA, USA) according to the manufacturer’s instructions. After cluster generation, the library preparations of a total of 12 samples were sequenced on an Illumina HiSeq X Ten platform using a 150-bp paired-end module.

Whole-transcriptome sequencing data were filtered and mapped to the porcine genome. The differentially expressed gene-seq method was based on a Poisson distribution (fold change >1.5 and adjusted *P*-value <0.05). Additive genes and synergistic genes (ASGs) were screened according to the definition. The whole-transcriptome sequencing data can be found in NCBI (https://dataview.ncbi.nlm.nih.gov/object/PRJNA779585?reviewer=3areeogpjo6vkvlr0i3hhb1s64) under accession number PRJNA779585.

According to the Kyoto Encyclopedia for Genes and Genomes (KEGG) annotation results and the official classification, we separately classified the functional and biological pathways of the differentially expressed genes and used R software for enrichment analysis. Bubble charts, heat maps, and volcano maps were generated to visualize the differentially expressed genes on the basis of log-normalized expression values of significant genes using v3 R software.

### Definition of Additive or Synergistic Genes

The additive genes and synergistic genes were defined according to a previous report (15). A gene was considered to be synergistically induced by IL-17/TNF-α when the combined effect was greater than the sum of the separate effects. A gene was considered to be additive when the combined effect was greater than the two individual effects but lower than the sum of the separate effects. In brief, we defined (*x*) to represent the log_2_-FCH (fold change) induced in hIL-17-treated PAECs (*y*) to represent the log_2_-FCH induced by hTNF-α and (*x* + *y*) to represent the log_2_-FCH induced by the combination of hIL-17 and hTNF-α in treated PAECs. Two tests were then used to define whether a gene was synergistic or additive, and the gene was excluded if it was antagonistic and induced by hIL-17 and hTNF-α. The flow chart was shown in **Supplementary Figure S1**. First, we used hypothesis testing to test whether the effect regulated by the two cytokines together was different than the sum of the individual effects, *i*.*e*.:


Test 1: Ho:(x+y)=(x)+(y) vs. Ha:(x+y)≠(x)+(y)

Then, we tested whether the combined effect was different than both individual effects, *i*.*e*,:


Test 2:Ho:(x+y)=(x) vs. Ha:(x+y)≠(x) and Ho:(x+y)=(y) vs. Ha:(x+y)≠(y)

### Synergistic Genes

A gene was considered synergistic if it passed test 1 and |(*x* + *y*)| > |(*x*) + (*y*)|. A synergistic increase is the difference |(*x* + *y*)| - |(*x*) + (*y*)|. Synergism can be either positive (if the synergist increase is positive) or negative.

### Additive Genes

A gene was considered additive (if it is not synergistic) if the combined effect was greater than both individual effects, *i*.*e*., the gene passes test 2 for both *x* and *y*.

The synergistic genes are highlighted in red, and the additive genes are shown in black in **Supplementary Tables S2, S3** (Ho: the null hypothesis; Ha: the alternative hypothesis).

### Statistical Analysis

Experimental data are presented as mean ± SEM. GraphPad Prism 5 software is used to perform statistical analysis and graph development. Statistical significance between the groups was calculated using two-tailed Student’s *t*-test using Microsoft Office Excel software. *p*-values <0.05 were considered significant.

## Results

### Human IL-17 and TNF-α Synergistically Regulated the Expression of Various Immune-Related Genes in PAECs

**Figure 1:**
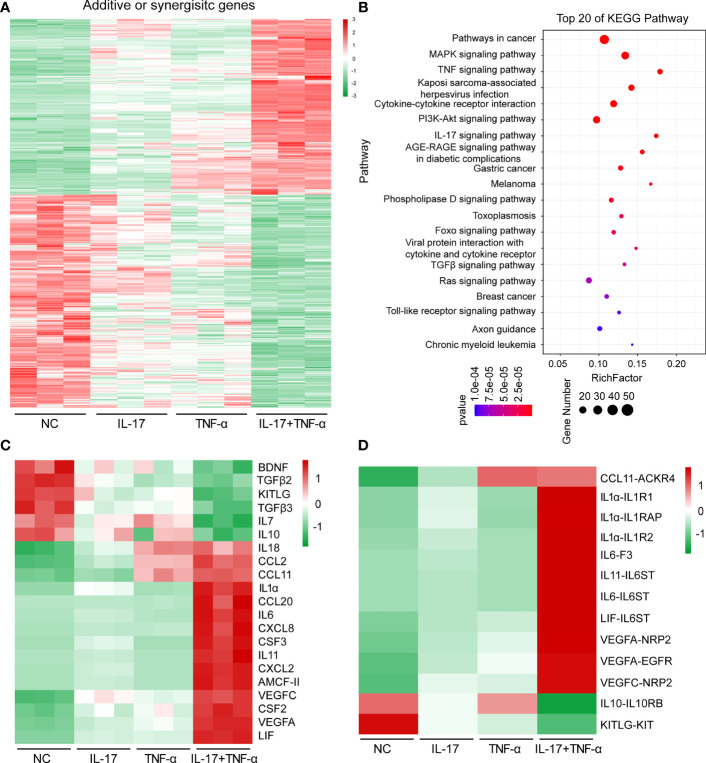
Human IL-17 and TNF-α additively or synergistically induced the expression of hundreds of genes in porcine aortic endothelial cells. **(A)** Heat map of additive or synergistic genes (ASGs) in the control group, IL-17 group, TNF-α group, and IL-17 plus TNF-α group. **(B)** Top 20 enriched Kyoto Encyclopedia for Genes and Genome pathways of ASGs between the control group and the IL-17 plus TNF-α group. **(C)** Heat map of regulated cytokine or chemokine gene expression levels in the control group, IL-17 group, TNF-α group, and IL-17 plus TNF-α group. **(D)** Ligand and receptor analysis of ASGs in the control group, IL-17 group, TNF-α group, and IL-17 plus TNF-α group.

**Figure 2:**
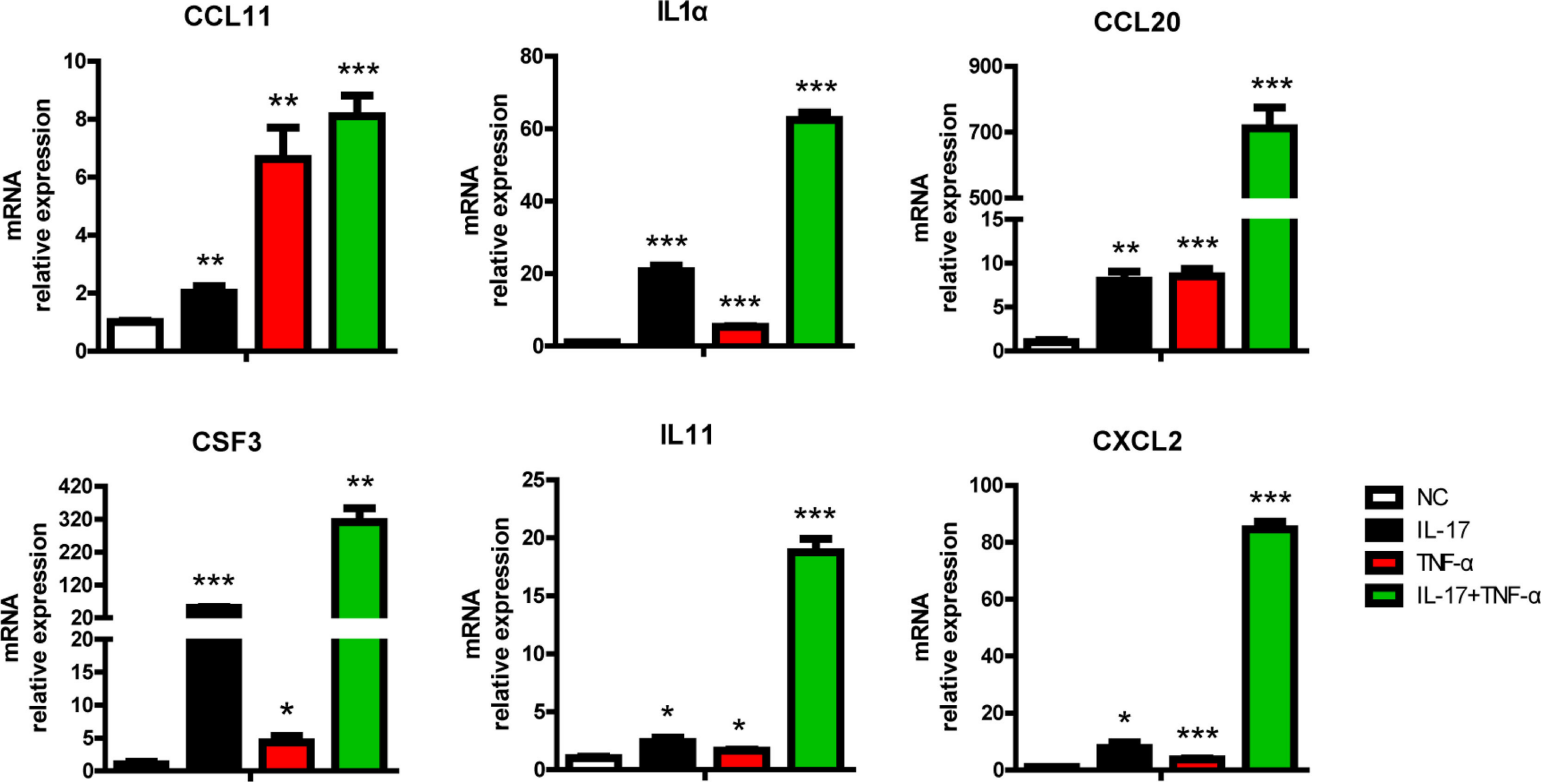
IL-17 and TNF-α additively or synergistically induced chemokine or cytokine expression in porcine aortic endothelial cells (PAECs). PAECs were treated with rhIL-17 (100 ng/ml), rhTNF-α (2 ng/ml), or rhIL-17 plus rhTNF-α for 0 or 6 h. The induction of CCL11, IL1α, CCL20, CSF3, IL11, and CXCL2 mRNA was measured *via* real-time PCR. Data are representative of at least three independent experiments (mean ± SEM). *p < 0.05, **p < 0.01, ***p < 0.001, determined by Student’s *t*-test.

Human IL-17 plus hTNF-α additively or synergistically regulated the expression of 697 genes in PAECs (**Figure 1A**, **Supplementary Tables S2, S3**). A total of 315 genes were upregulated in PAECs, 166 of which were synergistically induced, while another 149 genes were additively induced (**Supplementary Table S2**). A total of 382 genes were downregulated in PAECs, 249 of which were synergistically regulated, while 133 other genes were additively regulated (**Supplementary Table S3**). These ASGs were subjected to KEGG pathway enrichment analysis and annotation. Many immune-related signaling pathways, such as the TNF-α, IL-17, MAPK, and Toll-like receptor signaling pathways, were obviously enriched (**Figure 1B**). In addition to these pathways, the cytokine–cytokine receptor interaction was also enriched (**Figure 1B**). The KEGG pathway annotation analysis demonstrated that 159 genes were related to signal transduction (**Supplementary Figure S2**). Among the organismal systems, the immune system represented the top enrichment, involving 86 genes (**Supplementary Figure S2**), suggesting that hIL-17 plus hTNF-α primarily activated immune-related genes in PAECs. Cytokines and chemokines play critical roles in xenograft rejection, and we found that various proinflammatory cytokines, such as IL1α and IL6, and chemokines, including CCL2, CCL11, CXCL8, and CXCL2, were upregulated (**Figure 1C**). In contrast, the anti-inflammatory gene IL10 was downregulated (**Figure 1C**). The ligand–receptor analysis identified that CCL11, IL1α, IL6, IL11, and their receptors were upregulated, while IL-10-IL10RB and KITLG-KIT were obviously downregulated (**Figure 1D**). We used RT-PCR to validate the expression of several cytokines and chemokines and found that CCL20, CSF3, IL11, and CXCL2 were slightly induced by IL-17 or TNF-α alone but dramatically induced by IL-17 plus TNF-α, suggesting that these genes were synergistically induced (**Figure 2**). CCL11 and IL1α were additively induced by IL-17 plus TNF-α (**Figure 2**). These results were consistent with the transcriptome sequencing data (**Supplementary Table S2**). Collectively, the data suggest that hIL-17 and hTNF-α synergistically induce proinflammatory cytokine and chemokine expression to amplify the inflammatory response.

Due to several gene-modified pigs used for xenotransplantation, we asked whether human IL-17 and TNF-α could synergistically or additively induce proinflammatory cytokine and chemokine expression in PAECs from gene-modified pigs. We isolated PAECs from α1,3-galactosyltransferase (GGTA1) and cytidine monophosphate-N-acetylneuraminic acid hydroxylase (CMAH) gene double-deficient pigs. The two genes are responsible for the synthesis of Galα-1,3-Gal (Gal) and N-glycolylneuraminic acid (Neu5Gc), two carbohydrate xenoantigens that are respectively important for xenotransplantation, and the *GGTA1/CMAH* DKO pigs could largely reduce immune rejection in xenotransplantation. We treated the *GGTA1/CMAH DKO* PAECs with hIL-17, hTNF-α, or hIL-17 plus hTNF-α and found that IL-17 plus TNF-α synergistically induced the expression of CCL20, CSF3, and CXCL2 and additively induced the expression of CCL11, IL1α, and CXCL8 (**Supplementary Figure S3**). The data suggest that human IL-17 and TNF-α synergistically or additively induce proinflammatory cytokine or chemokine expression not only in wild-type PAECs but also in *GGTA1/CMAH DKO* PAECs. Thus, IL-17 and TNF-α likely promote the rejection of *GGTA1/CMAH DKO* xenograft in xenotransplantation.

We also found that IL-17 and TNF-α additively or synergistically regulate the expression of 65 cell surface proteins (**Supplementary Figure S4**): 29 genes were upregulated, and 36 genes were downregulated. The regulated genes included several receptors, adhesion molecules, and tight junction genes, such as IL1R1, ICAM1, and occludin (OCLN). These genes might increase proinflammatory signaling, promote immune cell migration, and enhance xenoantibody-mediated complement-dependent cytotoxicity (CDC). Moreover, some of these regulated cell surface genes might be xenoantigens, which will be investigated in the future.

### IL-17 Plus TNF-α Increased Neutrophil and Monocyte Chemotaxis

**Figure 3:**
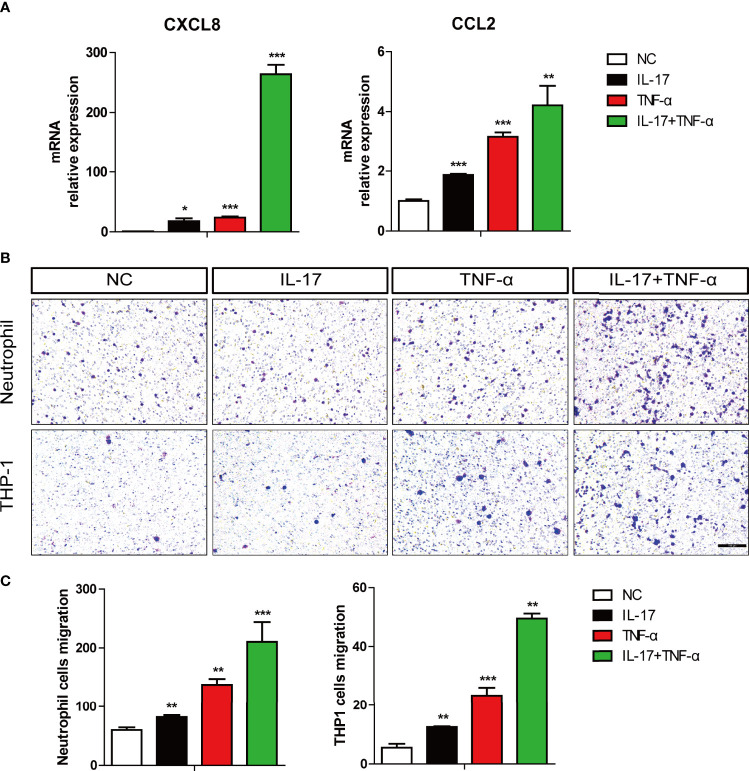
The combination of IL-17 with TNF-α largely increased the chemotaxis of THP-1 cells and human neutrophils. **(A)** Porcine aortic endothelial cells (PAECs) were treated with rhIL-17 (100 ng/ml), rhTNF-α (2 ng/ml), or rhIL-17 plus rhTNF-α for 0 or 6 h. The induction of CXCL8 or CCL2 mRNA was measured *via* real-time PCR. **(B)** The PAECs were treated with IL-17, TNF-α, or IL-17 plus TNF-α for 48 h, and the supernatant was collected for chemotaxis assays. Human neutrophils or THP‐1 cells were used to assess cell migration. **(C)** The number of migrating cells per field was determined as in **(B)**. Data are representative of at least three independent experiments (mean ± SEM). *p < 0.05, **p < 0.01, ***p < 0.001, determined by Student’s *t*-test.

The transcriptome sequencing data and our previous study showed that hIL-17 and hTNF-α synergistically induce CXCL8 expression and additively induce CCL2 expression in PAECs **(** **Figure 1C** and **Supplementary Table S2**) (12). We also validated their expression levels *via* RT-PCR analysis (**Figure 3A**). The chemokines CXCL8 and CCL2 have neutrophil and monocyte chemotactic activity, respectively; thus, we asked whether the supernatant of hIL-17- or hTNF-α-treated PAECs had monocyte and neutrophil chemotactic activity. We found that the supernatant of hIL-17- or hTNF-α-treated PAECs alone increased human neutrophil migration and that the supernatant of hIL-17 plus hTNF-α-treated PAECs had greater chemotactic activity than the supernatant of IL-17- or TNF-α-treated PAECs alone (**Figures 3B, C** **)**. In a THP-1 (a human leukemia monocytic cell line) cell migration assay, the results were similar to those of human neutrophil migration (**Figures 3B, C** **)**. In order to investigate whether IL-17 or TNF-α directly mediated THP-1 cell migration, we collected the supernatant from PAECs without IL-17 or TNF-α treatment and then added IL-17 plus TNF-α to the supernatant for THP-1 cell migration assay. We found that the supernatant with IL-17 plus TNF-α did not enhance THP-1 cell migration, suggesting that IL-17 or TNF-α cannot directly mediate THP-1 cell migration (**Supplementary Figure S5**). To confirm whether IL-17 plus TNF-α increased THP-1 cell migration through inducing CCL2 expression, we used RS504393, a specific inhibitor of CCR2 which is the receptor for CCL2, treated THP-1 cells, and found that RS504393 almost completely blocked THP-1 cell migration, which suggest that IL-17 plus TNF-α increases THP-1 cell chemotaxis through enhancing CCL2 expression (**Supplementary Figure S5**). Collectively, these data suggest that hIL-17 plus hTNF-α increases human neutrophil or monocyte chemotaxis and that the increased chemotaxis is likely due to increased CXCL8 or CCL2 production by PAECs.

### IL-17 Plus TNF-α Regulated Coagulation-Related Gene Expression in PAECs

**Figure 4:**
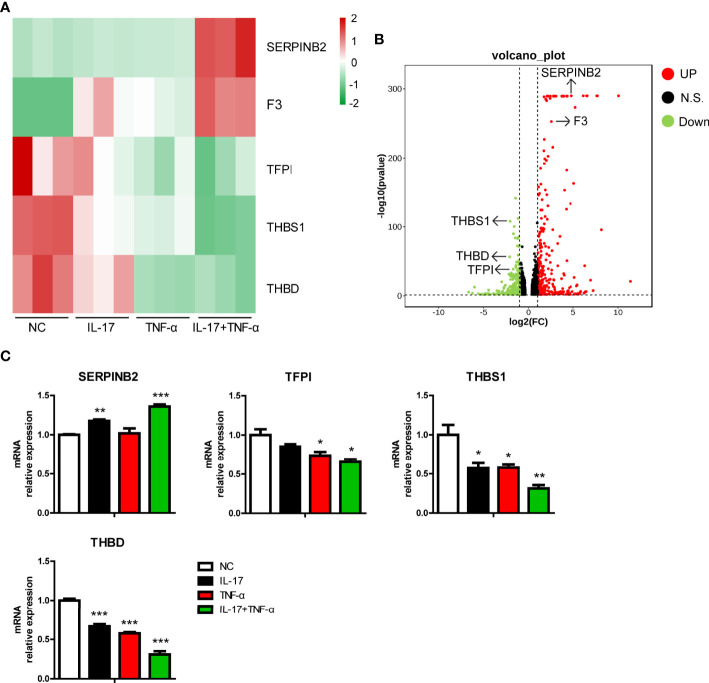
IL-17 and TNF-α regulated coagulation-related gene expression in porcine aortic endothelial cells (PAECs). **(A)** Heat map showing regulated coagulation-related additive or synergistic genes (ASGs) in the control group, IL-17 group, TNF-α group, and IL-17 plus TNF-α group. **(B)** Volcano plots displaying regulated coagulation-related ASGs between the control group and the IL-17 plus TNF-α group. **(C)** The PAECs were treated with rhIL-17 (100 ng/ml), rhTNF-α (2 ng/ml), or rhIL-17 plus rhTNF-α for 0 or 6 h. The induction of SERPINB2, TFPI, THBS1, and THBD mRNA was measured *via* real-time PCR. Data are representative of at least three independent experiments (mean ± SEM). *p < 0.05, **p < 0.01, ***p < 0.001, determined by Student’s *t*-test.

Previously, we found that hIL-17 plus hTNF-α increased the mRNA level of F3, a procoagulation gene, in PAECs (12). In the present study, we found that F3 was induced in PAECs treated with hIL-17 plus hTNF-α (**Figure 4A**). In addition to F3, we found that the mRNA level of another pro-coagulation gene (SERPINB2) was upregulated, while the mRNA levels of three anti-coagulation genes (TFPI, THBS1, and THBD) were downregulated in PAECs treated with hIL-17 plus hTNF-α (**Figures 4A, B** **)**. To validate the data, we measured the mRNA levels of these genes in PAECs treated with hIL-17 or hTNF-α and found that SERPINB2 was increased, while TFPI, THBS1, and THBD were downregulated in PAECs treated with hIL-17 plus hTNF-α (**Figure 4C**). The mRNA levels of THBS1 and THBD were slightly downregulated by IL-17 or TNF-α alone and largely reduced by IL-17 plus TNF-α. Collectively, these data suggest that hIL-17 and hTNF-α amplify the coagulation reaction in response to xenografts through additive or synergistic effects.

### IL-17 Plus TNF-α Promoted Human Antibody-Mediated Complement-Dependent Cytotoxicity in PAECs

**Figure 5:**
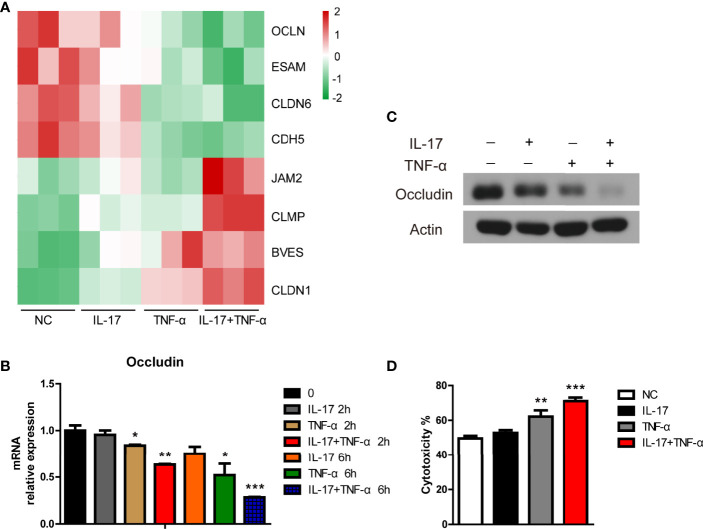
IL-17 increased TNF-α-mediated cytotoxicity toward porcine aortic endothelial cells (PAECs) in a human antibody-mediated complement-dependent cytotoxicity model. **(A)** Heat map showing tight junction genes among the additive or synergistic genes in the control group, IL-17 group, TNF-α group, and IL-17 plus TNF-α group. **(B)** The PAECs were treated with rhIL-17 (100 ng/ml), rhTNF-α (2 ng/ml), or rhIL-17 plus rhTNF-α for 0, 2, or 6 h. The mRNA level of occludin was measured *via* real-time PCR. **(C)** The PAECs were treated with rhIL-17 (100 ng/ml), rhTNF-α (2 ng/ml), or rhIL-17 plus rhTNF-α for 24 h. The lysates were analyzed by western blotting with antibodies against occludin and actin. **(D)** The PAECs were treated with rhIL-17 (100 ng/ml), rhTNF-α (2 ng/ml), or rhIL-17 plus rhTNF-α for 24 h and then exposed to human serum to induce antibody-mediated CDC. Data are representative of at least three independent experiments (mean ± SEM). *p < 0.05, **p < 0.01, ***p < 0.001, determined by Student’s *t*-test.

Tight junction genes play an important role in xenoantibody-mediated CDC (20, 21). In the present study, we found that OCLN, ESAM, CLDN6, and CDH5 were downregulated and that JAM2, CLMP, BVES, and CLDN1 were upregulated in PAECs treated with hIL-17 plus hTNF-α (**Figure 5A**). Moreover, we found that hTNF-α alone decreased OCLN expression in PAECs and that the mRNA and protein levels of occludin were much lower in PAECs treated with hIL-17 plus hTNF-α than in PAECs treated with TNF-α alone (**Figures 5B, C** **)**. We previously found that hTNF-α promoted human antibody-mediated CDC in porcine ECs by downregulating occludin expression; thus, we asked whether hIL-17 plus hTNF-α had stronger PAEC cytotoxicity in a human-mediated CDC model. We found that TNF-α alone exerted increased PAEC cytotoxicity, while hIL-17 plus hTNF-α led to more PAEC death than TNF-α alone (**Figure 5D**). To exclude the direct cytotoxicity of hIL-17 or hTNF-α to PAECs, we treated PAECs with or without hIL-17, hTNF-α, or hIL-17 plus hTNF-α for 48 h and assess the viability of PAECs with CCK8. We found that the OD450 of the negative control group was almost equal to hIL-17, hTNF-α, or hIL-17 plus the hTNF-α treated group, suggesting that hIL-17 or hTNF-α does not directly affect the viability of PAECs (**Supplementary Figure S6**). These data suggest that hIL-17 and hTNF-α likely increase xenoantibody-mediated CDC, which leads to xenograft injury.

### IL-17 Decreased TNF-α-Mediated SLA-I Upregulation in PAECs

**Figure 6:**
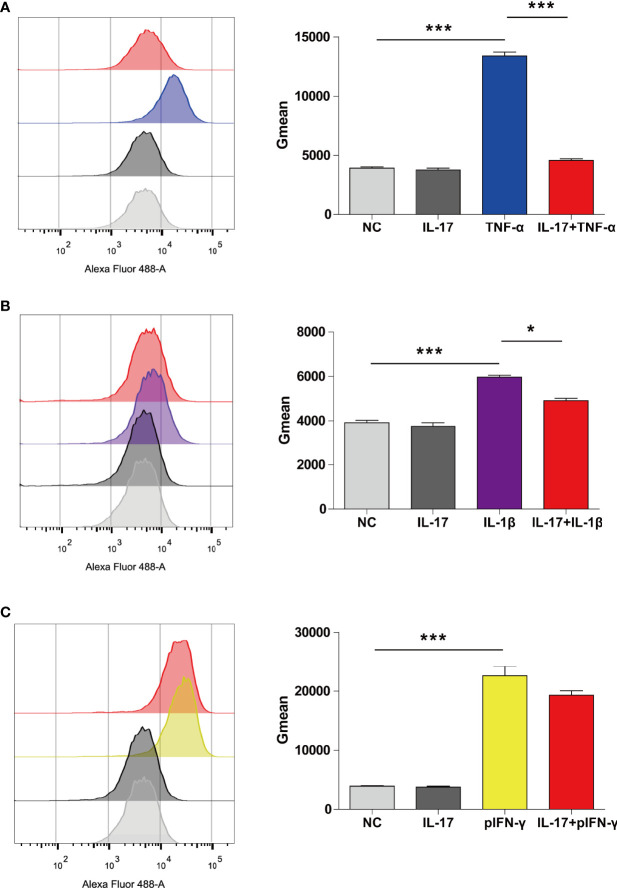
IL-17 decreased TNF-α- or IL-1β-induced SLA-I expression in porcine aortic endothelial cells (PAECs). **(A)** The PAECs were untreated or treated with rhIL-17 (100 ng/ml), rhTNF-α (2 ng/ml) or rhIL-17 plus rhTNF-α for 24 h, and the expression of SLA-I was measured *via* flow cytometry. **(B)** The PAECs were untreated or treated with rhIL-17 (100 ng/ml), rhIL-1β (20 ng/ml) or rhIL-17 plus rhIL-1β for 24 h, and the expression of SLA-I **(A)** was measured *via* flow cytometry. **(C)** The PAECs were untreated or treated with rhIL-17 (100 ng/ml), rpIFN-γ (40 ng/ml) or rhIL-17 plus rpIFN-γ for 24 h, and the expression of SLA-I was measured *via* flow cytometry. The degree of SLA-I binding to PAECs was evaluated by determining the geometric mean fluorescence intensity (Gmean). Data are representative of at least three independent experiments (mean ± SEM). *p < 0.05, ***p < 0.001, determined by Student’s *t*-test.

We previously found that hTNF-α could induce SLA-I expression; therefore, we asked whether hIL-17 plus hTNF-α could additively or synergistically increase SLA-I expression (12). Surprisingly, we found that hTNF-α significantly induced SLA-I expression; however, hIL-17 almost completely blocked hTNF-α-mediated SLA-I upregulation (**Figure 6A**). We also found that hIL-1β increased SLA-I expression and that hIL-17 slightly reduced hIL-1β-mediated SLA-I upregulation (**Figure 6B**). Moreover, porcine IFN-γ (pIFN-γ) increased SLA-I expression, but IL-17 did not decrease IFN-γ-mediated SLA-I expression (**Figure 6C**). These data suggest that IL-17 has a suppressive effect on TNF-α- or IL-1β-mediated SLA-I upregulation.

### IL-17 and IL-1β or IFN-γ Had Additive or Synergistic Effects in PAECs

**Figure 7:**
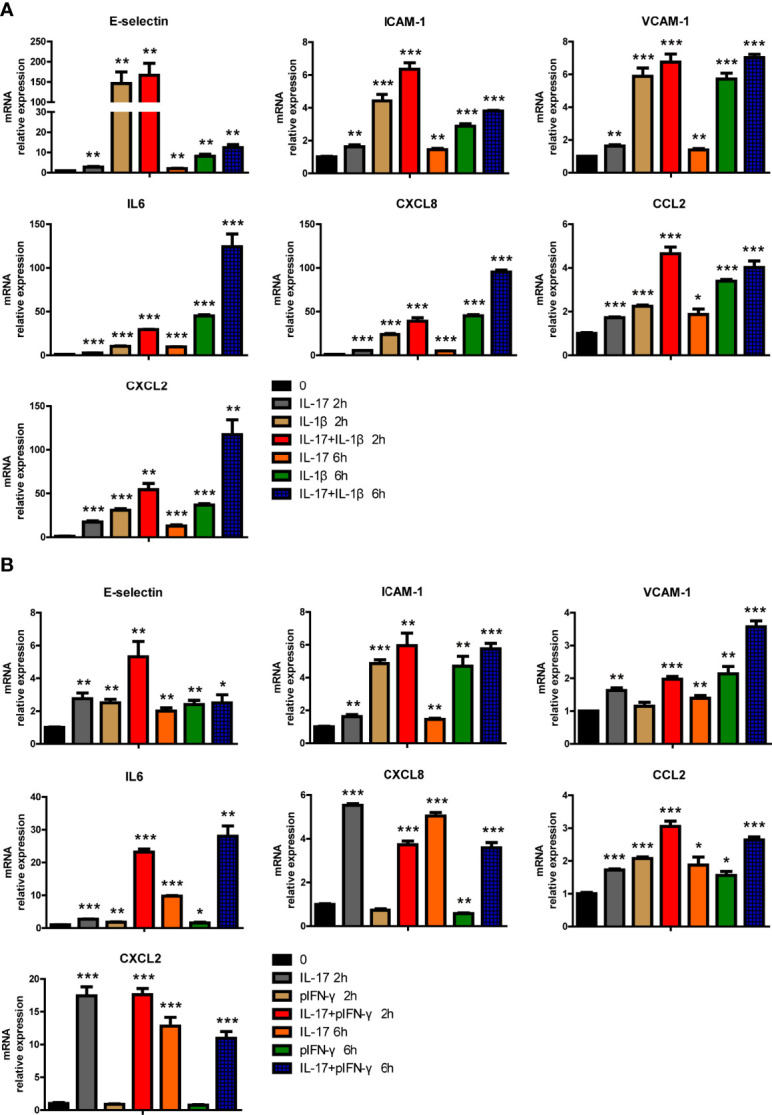
IL-17 and IL-1β or IFN-γ additively or synergistically induced the expression of certain proinflammatory genes in porcine aortic endothelial cells (PAECs). **(A)** The PAECs were treated with rhIL-17 (50 ng/ml), rhIL-1β (10 ng/ml), or rhIL-17 plus rhIL-1β for 0, 2, or 6 h. The induction of E-selectin, VCAM-1, ICAM-1, IL-6, IL-8, MCP-1, or CXCL2 mRNA was measured using real-time PCR. **(B)** The PAECs were treated with rhIL-17 (50 ng/ml), rpIFN-γ (40 ng/ml), or rhIL-17 plus rpIFN-γ for 0, 2, or 6 h. The induction of E-selectin, VCAM-1, ICAM-1, IL-6, IL-8, MCP-1, or CXCL2 mRNA was measured using real-time PCR. Data are representative of at least three independent experiments (mean ± SEM). *p < 0.05, **p < 0.01, ***p < 0.001, determined by Student’s *t*-test.

Human IL-17 and hTNF-α additively or synergistically regulated the expression of various genes in PAECs, and we wondered whether hIL-17, in combination with hIL-1β or pIFN-γ, had additive or synergistic effects in PAECs. We found that hIL-17 and hIL-1β synergistically induced IL6 expression and additively induced E-selectin, ICAM-1, VCAM-1, CXCL8, CCL2, and CXCL2 expression (**Figure 7A**). Human IL-17 and pIFN-γ also synergistically induced IL6 expression and additively induced E-selectin, ICAM-1, VCAM-1, and CCL2 expression (**Figure 7B**). However, human IL-17 and pIFN-γ did not additively or synergistically induce CXCL8 or CXCL2 expression (**Figure 7B**). These data suggest that hIL-17 and hIL-1β or pIFN-γ can additively or synergistically induce the expression of specific proinflammatory cytokines, chemokines, and adhesion genes in PAECs.

## Discussion

We previously found that hIL-17 and hTNF-α can activate PAECs and induce downstream gene expression in PAECs (12). However, the pathological role of hIL-17 and hTNF-α in xenotransplantation is less well investigated. Here we found that hIL-17 and hTNF-α additively and synergistically regulate the expression of 697 genes in PAECs: 415 genes were synergistically regulated, and 282 genes were additively regulated. We found that hIL-17 plus hTNF-α increased the expression of many proinflammatory cytokines and chemokines and reduced the expression of specific anti-inflammatory genes. Moreover, we found that hIL-17 plus hTNF-α promoted human neutrophil and THP-1 migration by inducing CXCL8 and CCL2 expression. Human IL-17 plus hTNF-α increased procoagulation gene expression and decreased anti-coagulation gene expression. Human IL-17 plus hTNF-α increased human antibody-mediated CDC in PAECs. Based on our observations, we speculate that hIL-17 and hTNF-α have important pathological roles in promoting inflammation, the coagulation response, and xenoantibody-mediated cell injury in pig-to-primate xenotransplantation.

Several studies have reported that IL-17 and TNF-α have additive and synergistic effects in mouse and human systems (15, 16, 22, 23). The synergistic effect has been reported to promote the development of immune-related diseases, such as psoriasis and rheumatoid arthritis (15, 16). Here we found that hIL-17 and hTNF-α synergistically regulate the expression of 415 genes in PAECs. The KEGG pathway annotation suggested that most of these genes are associated with the immune system. Human IL-17 and hTNF-α synergistically increased proinflammatory gene (such as CCL20, IL11, IL6, CXCL2, and CXCL8) expression and reduced anti-inflammatory gene (such as IL10) expression. Moreover, some receptors for these genes were also additively or synergistically regulated. The expression of IL6ST (receptor for IL11 or IL6) or F3 (receptor for IL6) was induced, while the expression of IL10RB (receptor for IL10) was decreased in PAECs treated with hIL-17 plus hTNF-α. These data suggest that hIL-17 and hTNF-α might amplify the immune response by synergistically increasing proinflammatory gene expression and decreasing anti-inflammatory gene expression in pig-to-human xenotransplantation.

The chemokines CXCL8 and CCL2 can recruit neutrophils and monocytes to the inflammatory site, respectively (24–27). The present study found that hIL-17 and hTNF-α synergistically increased CXCL8 expression and additively induced CCL2 expression in PAECs. The supernatant from hIL-17 plus hTNF-α-treated PAECs had much more chemotactic activity (to recruit human neutrophils and THP-1 cells) than the supernatant from hIL-17- or hTNF-α-treated PAECs. Thus, our study suggests that hIL-17 plus hTNF-α has the potential ability to recruit neutrophils and monocytes to inflammatory response sites to amplify the immune reaction.

The coagulation cascade is a tightly regulated process (28). In pig-to-primate xenotransplantation, the coagulation cascade is dysregulated, and this dysregulation of the coagulation cascade is a major obstacle for xenograft survival (29). F3 promotes the conversion of prothrombin to thrombin to initiate the extrinsic coagulation cascade (30). Previously, we found that hIL-17 plus hTNF-α increases F3 expression (12). In the present study, we found that hIL-17 plus hTNF-α significantly increased the expression of two procoagulation factors (SERPINB2 and F3) and decreased the expression of three anticoagulation factors (TFPI, THBS1, and THBD). Based on these data, we speculate that hIL-17 and hTNF-α likely promote the coagulation cascade and consequently decrease xenograft survival in xenotransplantation. To confirm the role of hIL-17 and hTNF-α in the coagulation response in xenotransplantation, we intend to design experiments to answer this question in the near future.

Xenoantibody-mediated complement-dependent cell killing is another limiting factor in xenograft survival. Human antibody-mediated CDC is a suitable *in vitro* model to mimic the process. The barrier function of porcine ECs is important for the protection of ECs in human antibody-mediated CDC. Tight junction genes are critical for barrier integrity (31). Dalmasso et al. reported that IL-4 protected porcine ECs from human antibody-mediated CDC by partially increasing claudin 5 expression (20). We previously found that TNF-α promoted porcine EC killing by decreasing occludin expression (21). Moreover, claudin 2 protected porcine ECs from human antibody-mediated CDC (21). The present study found that hIL-17 plus hTNF-α decreased the expression of four tight junction genes and increased the expression of four tight junction genes. We also found that hIL-17 plus hTNF-α obviously decreased the mRNA and protein levels of occludin. As expected, hIL-17 plus hTNF-α increased the cytotoxicity toward PAECs in human antibody-mediated CDC. In addition to occludin, whether other regulated tight junction genes contribute to IL-17 plus TNF-α-mediated cytotoxicity promotion in human antibody-mediated CDC needs to be investigated.

T-cell response is important for the cellular immune response to a xenograft (32). SLA-I is primarily responsible for CD8^+^ T-cell activation. Previously, we found that TNF-α and IL-1β increased the expression of SLA-I in PAECs (12). In the present study, we found similar results. Interestingly, we found that hIL-17 almost completely blocked hTNF-α-mediated SLA-I upregulation. Human IL-17 also partially inhibited hIL1β-mediated SLA-I expression. However, hIL-17 did not suppress porcine IFN-γ-induced SLA-I expression. The data suggest that hIL-17 differentially regulates TNF-α-, IL-1β-, or IFN-γ-induced SLA-I expression, and the detailed molecular mechanism needs to be investigated in the future. Human IL-17 might suppress CD8^+^ T-cell-mediated cell killing in xenotransplantation. In addition to IL-17 and TNF-α, IL-17 and IL-1β or IFN-γ also had additive or synergetic effects, and whether IL-17 and IL-1β or IFN-γ also play pathological roles in xenotransplantation needs to be investigated.

Regarding the additive or synergistic effect of hIL-17 and hTNF-α, the combined inhibition of IL-17 and TNF-α has additive or synergistic effects in the therapy of certain diseases. In rheumatoid arthritis, a previous report found that the combined inhibition of IL-17 and TNF-α was effective in blocking tissue destruction associated with arthritis (16, 33). Moreover, the combined blockade of IL-17 and IL-1β showed beneficial synergistic effects to prevent joint inflammation, cartilage destruction, and bone damage in a collagen-induced arthritis mouse model (34). These studies suggest that the combined blockade of hIL-17 and hTNF-α might have superior efficacy over anti-IL-17 or anti-TNF-α blockade alone.

In conclusion, in the present study, we found that (i) hIL-17 and hTNF-α synergistically induced the expression of hundreds of genes in PAECs, (ii) hIL-17 and hTNF-α additively or synergistically induced the expression of various proinflammatory genes and certain anti-inflammatory factors, and (iii) hIL-17 plus hTNF-α promoted immune cell migration and human antibody-mediated CDC, increased procoagulation gene expression, and inhibited anticoagulation gene expression. Further *in vivo* experiments are needed to confirm these pathological roles in xenotransplantation. Overall, coblockade of IL-17 and TNF-α might be a promising way to increase xenograft survival.

## Data Availability Statement

The datasets presented in this study can be found in online repositories. The names of the repository/repositories and accession number(s) can be found in the article/**Supplementary Material**.

## Ethics Statement

The studies involving human participants were reviewed and approved by the Institutional Biomedical Research Ethics Committee of the Guangdong Medical University. The patients/participants provided their written informed consent to participate in this study.

## Author Contributions

HG, XC, SL, and LM designed the experiments. HG wrote the manuscript. HG, WL, PC, YZ, and MC conducted the experiments and analyzed the data. WH, LP, HS, DH, HW, ZS, and HZ helped with the experiments. WL and PC analyzed the transcriptome sequencing data. HG and XC supervised the study.

## Funding

This work was supported by grants from the National Natural Science Foundation of China (grant/award numbers 81902021, 81902558, and 81801474), Guangdong Basic and Applied Basic Research Foundation (grant/award numbers 2019A1515011009, 2021A1515010683, 2020A1515010225, and 2021A1515010955), Shenzhen Foundation of Science and Technology (grant/award numbers JCYJ20180306172449376, JCYJ20180306172459580, JCYJ20180306172419505, and JCYJ20180306172502097), Shenzhen Longhua District Foundation of Science and Technology (grant/award number SZLHQJCYJ202002), the Scientific Research Projects of Medical and Health Institutions of Longhua District, Shenzhen (2021014), Longhua District Key Laboratory of Genomics and Precision Medicine (grant/award number 20170913A0410026), and Shenzhen Longhua District Key Laboratory for diagnosis and treatment of chronic kidney disease(grant/award number 2022-7).

## Conflict of Interest

The authors declare that this research was conducted in the absence of any commercial or financial relationships that could be construed as a potential conflict of interest.

## Publisher’s Note

All claims expressed in this article are solely those of the authors and do not necessarily represent those of their affiliated organizations, or those of the publisher, the editors and the reviewers. Any product that may be evaluated in this article, or claim that may be made by its manufacturer, is not guaranteed or endorsed by the publisher.

## References

[1] Lu T Yang B Wang R Qin C. Xenotransplantation: Current Status in Preclinical Research. Front Immunol (2019) 10:3060. doi: 10.3389/fimmu.2019.03060 32038617 PMC6989439

[2] Cooper DKC Hara H Iwase H Yamamoto T Wang ZY Jagdale A. Pig Kidney Xenotransplantation: Progress Toward Clinical Trials. Clin Transplant (2021) 35(1):e14139. doi: 10.1111/ctr.14139 33131148

[3] Reichart B Langin M Radan J Mokelke M Buttgereit I Ying J. Pig-To-non-Human Primate Heart Transplantation: The Final Step Toward Clinical Xenotransplantation? J Heart Lung Transplant Off Publ Int Soc Heart Transplant (2020) 39(8):751–7. doi: 10.1016/j.healun.2020.05.004 32527674

[4] Cooper DKC Ekser B Tector AJ. Immunobiological Barriers to Xenotransplantation. Int J Surg (2015) 23(Pt B):211–6. doi: 10.1016/j.ijsu.2015.06.068 PMC4684773 26159291

[5] Lai L Kolber-Simonds D Park KW Cheong HT Greenstein JL Im GS. Production of Alpha-1,3-Galactosyltransferase Knockout Pigs by Nuclear Transfer Cloning. Science (2002) 295(5557):1089–92. doi: 10.1126/science.1068228 11778012

[6] Phelps CJ Koike C Vaught TD Boone J Wells KD Chen SH. Production of Alpha 1,3-Galactosyltransferase-Deficient Pigs. Science (2003) 299(5605):411–4. doi: 10.1126/science.1078942 PMC3154759 12493821

[7] Yang L Guell M Niu D George H Lesha E Grishin D. Genome-Wide Inactivation of Porcine Endogenous Retroviruses (PERVs). Science (2015) 350(6264):1101–4. doi: 10.1126/science.aad1191 26456528

[8] Niu D Wei HJ Lin L George H Wang T Lee IH. Inactivation of Porcine Endogenous Retrovirus in Pigs Using CRISPR-Cas9. Science (2017) 357(6357):1303–7. doi: 10.1126/science.aan4187 PMC5813284 28798043

[9] Yue Y Xu W Kan Y Zhao HY Zhou Y Song X. Extensive Germline Genome Engineering in Pigs. Nat Biomed Eng (2021) 5(2):134–43. doi: 10.1038/s41551-020-00613-9 32958897

[10] Naeimi Kararoudi M Hejazi SS Elmas E Hellstrom M Naeimi Kararoudi M Padma AM. Clustered Regularly Interspaced Short Palindromic Repeats/Cas9 Gene Editing Technique in Xenotransplantation. Front Immunol (2018) 9:1711. doi: 10.3389/fimmu.2018.01711 30233563 PMC6134075

[11] Gao H Chen P Wei L Xu J Liu L Zhao Y. Angiopoietin-1 and Angiopoietin-2 Protect Porcine Iliac Endothelial Cells From Human Antibody-Mediated Complement-Dependent Cytotoxicity Through Phosphatidylinositide 3-Kinase/AKT Pathway Activation. Xenotransplantation (2017) 24(4). doi: 10.1111/xen.12309 28474373

[12] Gao H Liu L Zhao Y Hara H Chen P Xu J. Human IL-6, IL-17, IL-1beta, and TNF-Alpha Differently Regulate the Expression of Pro-Inflammatory Related Genes, Tissue Factor, and Swine Leukocyte Antigen Class I in Porcine Aortic Endothelial Cells. Xenotransplantation (2017) 24(2). doi: 10.1111/xen.12291 28303603

[13] Pober JS Sessa WC. Evolving Functions of Endothelial Cells in Inflammation. Nat Rev Immunol (2007) 7(10):803–15. doi: 10.1038/nri2171 17893694

[14] Zhao Y Cooper DKC Wang H Chen P He C Cai Z. Potential Pathological Role of Pro-Inflammatory Cytokines (IL-6, TNF-Alpha, and IL-17) in Xenotransplantation. Xenotransplantation (2019) 26(3):e12502. doi: 10.1111/xen.12502 30770591

[15] Chiricozzi A Guttman-Yassky E Suarez-Farinas M Nograles KE Tian S Cardinale I. Integrative Responses to IL-17 and TNF-Alpha in Human Keratinocytes Account for Key Inflammatory Pathogenic Circuits in Psoriasis. J Invest Dermatol (2011) 131(3):677–87. doi: 10.1038/jid.2010.340 21085185

[16] Fischer JA Hueber AJ Wilson S Galm M Baum W Kitson C. Combined Inhibition of Tumor Necrosis Factor Alpha and Interleukin-17 as a Therapeutic Opportunity in Rheumatoid Arthritis: Development and Characterization of a Novel Bispecific Antibody. Arthritis Rheumatol (2015) 67(1):51–62. doi: 10.1002/art.38896 25303306

[17] Gao H Zhao C Xiang X Li Y Zhao Y Li Z. Production of Alpha1,3-Galactosyltransferase and Cytidine Monophosphate-N-Acetylneuraminic Acid Hydroxylase Gene Double-Deficient Pigs by CRISPR/Cas9 and Handmade Cloning. J Reprod Dev (2017) 63(1):17–26. doi: 10.1262/jrd.2016-079 27725344 PMC5320426

[18] Song X Gao H Lin Y Yao Y Zhu S Wang J. Alterations in the Microbiota Drive Interleukin-17C Production From Intestinal Epithelial Cells to Promote Tumorigenesis. Immunity (2014) 40(1):140–52. doi: 10.1016/j.immuni.2013.11.018 24412611

[19] Chen J Gao H Chen L Wang X Song Z Cooper DKC. A Potential Role of TLR2 in Xenograft Rejection of Porcine Iliac Endothelial Cells: An *In Vitro* Study. Xenotransplantation (2019) 26(5):e12526. doi: 10.1111/xen.12526 31127671

[20] Dalmasso AP Goldish D Benson BA Tsai AK Wasiluk KR Vercellotti GM. Interleukin-4 Induces Up-Regulation of Endothelial Cell Claudin-5 Through Activation of FoxO1: Role in Protection From Complement-Mediated Injury. J Biol Chem (2014) 289(2):838–47. doi: 10.1074/jbc.M113.455766 PMC3887209 24280217

[21] Gao H Cao M Chen P Cooper DKC Zhao Y Wei L. TNF-Alpha Promotes Human Antibody-Mediated Complement-Dependent Cytotoxicity of Porcine Endothelial Cells Through Downregulating P38-Mediated Occludin Expression. Cell Communicat Signaling CCS (2019) 17(1):75. doi: 10.1186/s12964-019-0386-7 PMC6631523 31307477

[22] Liu Y Mei J Gonzales L Yang G Dai N Wang P. IL-17A and TNF-Alpha Exert Synergistic Effects on Expression of CXCL5 by Alveolar Type II Cells *In Vivo* and *In Vitro* . J Immunol (2011) 186(5):3197–205. doi: 10.4049/jimmunol.1002016 21282514

[23] Griffin GK Newton G Tarrio ML Bu DX Maganto-Garcia E Azcutia V. IL-17 and TNF-Alpha Sustain Neutrophil Recruitment During Inflammation Through Synergistic Effects on Endothelial Activation. J Immunol (2012) 188(12):6287–99. doi: 10.4049/jimmunol.1200385 PMC3370121 22566565

[24] Qian BZ Li J Zhang H Kitamura T Zhang J Campion LR. CCL2 Recruits Inflammatory Monocytes to Facilitate Breast-Tumour Metastasis. Nature (2011) 475(7355):222–5. doi: 10.1038/nature10138 PMC3208506 21654748

[25] Conrady CD Zheng M Mandal NA van Rooijen N Carr DJ. IFN-Alpha-Driven CCL2 Production Recruits Inflammatory Monocytes to Infection Site in Mice. Mucosal Immunol (2013) 6(1):45–55. doi: 10.1038/mi.2012.46 22692455 PMC3449026

[26] Baggiolini M. CXCL8 - The First Chemokine. Front Immunol (2015) 6. doi: 10.3389/fimmu.2015.00285 PMC4459227 26106391

[27] Park JH Lee HK. Re-Analysis of Single Cell Transcriptome Reveals That the NR3C1-CXCL8-Neutrophil Axis Determines the Severity of COVID-19. Front Immunol (2020) 11:2145. doi: 10.3389/fimmu.2020.02145 32983174 PMC7485000

[28] Furie B Furie BC. Mechanisms of Thrombus Formation. New Engl J Med (2008) 359(9):938–49. doi: 10.1056/NEJMra0801082 18753650

[29] Iwase H Ezzelarab MB Ekser B Cooper DK. The Role of Platelets in Coagulation Dysfunction in Xenotransplantation, and Therapeutic Options. Xenotransplantation (2014) 21(3):201–20. doi: 10.1111/xen.12085 24571124

[30] Ahrens HE Petersen B Herrmann D Lucas-Hahn A Hassel P Ziegler M. siRNA Mediated Knockdown of Tissue Factor Expression in Pigs for Xenotransplantation. Am J Transplant Off J Am Soc Transplant Am Soc Transplant Surg (2015) 15(5):1407–14. doi: 10.1111/ajt.13120 25808638

[31] Huang ZQ Liu J Ong HH Yuan T Zhou XM Wang J. Interleukin-13 Alters Tight Junction Proteins Expression Thereby Compromising Barrier Function and Dampens Rhinovirus Induced Immune Responses in Nasal Epithelium. Front Cell Dev Biol (2020) 8:572749. doi: 10.3389/fcell.2020.572749 33102478 PMC7546404

[32] Higginbotham L Ford ML Newell KA Adams AB. Preventing T Cell Rejection of Pig Xenografts. Int J Surg (2015) 23(Pt B):285–90. doi: 10.1016/j.ijsu.2015.07.722 26306770

[33] Buckland J. Rheumatoid Arthritis: Anti-TNF and Anti-IL-17 Antibodies–Better Together! Nat Rev Rheumatol (2014) 10(12):699. doi: 10.1038/nrrheum.2014.183 25348041

[34] Zhang Y Ren G Guo M Ye X Zhao J Xu L. Synergistic Effects of Interleukin-1beta and Interleukin-17A Antibodies on Collagen-Induced Arthritis Mouse Model. Int Immunopharmacol (2013) 15(2):199–205. doi: 10.1016/j.intimp.2012.12.010 23280345

