# Corrigendum: Human IL-17 and TNF-α additively or synergistically regulate the expression of proinflammatory genes, coagulation-related genes, and tight junction genes in porcine aortic endothelial cells

**DOI:** 10.3389/fimmu.2024.1439283

**Published:** 2024-06-26

**Authors:** Weilong Li, Pengfei Chen, Yanli Zhao, Mengtao Cao, Wenjun Hu, Litao Pan, Huimin Sun, Dongsheng Huang, Hanxi Wu, Zhuoheng Song, Huanli Zhong, Lisha Mou, Shaodong Luan, Xiehui Chen, Hanchao Gao

**Affiliations:** ^1^ Department of Nephrology, Shenzhen Longhua District Central Hospital, Affiliated Central Hospital of Shenzhen Longhua District, Guangdong Medical University, Shenzhen, China; ^2^ Department of Anesthesiology, The 305 Hospital of People’s Liberation Army of China (PLA), Beijing, China; ^3^ Department of Acupuncture and Massage, Shenzhen Second People’s Hospital, The First Affiliated Hospital of Shenzhen University, Shenzhen, China; ^4^ Department of Medical Administration, People’s Hospital of Shenzhen Longhua Branch, Shenzhen, China

**Keywords:** xenotransplantation, immune rejection, cytokines, IL-17, TNF-α, PAECs, inflammation, coagulation

In the published article, there was an error in the Funding statement. We had missed writing a fund, the Scientific Research Projects of Medical and Health Institutions of Longhua District, Shenzhen (2021014). The correct Funding statement appears below:

