# Supplementary material for: Human IL-17 and TNF-α Additively or Synergistically Regulate the Expression of Proinflammatory Genes, Coagulation-Related Genes, and Tight Junction Genes in Porcine Aortic Endothelial Cells
Source: Front Immunol. 2022 Jun 30;13:857311. doi: 10.3389/fimmu.2022.857311 (PMC9279740; doi:10.3389/fimmu.2022.857311)
Supplement: Supplementary file 1 [file DataSheet_1.docx]

| Gene name | Primers sequence (5’-3’) |
| --- | --- |
| CCL11 | agaagacaacagctctccct |
|  | gtgagcagcagacacagaa |
| IL1α | gtgaagatggccaaagtccc |
|  | ccaggaagtggctcatagct |
| CCL20 | cccagcactgagcacatcta |
|  | tgctgcttctgacttgctgc |
| CSF3 | tggcacattgcactctggat |
|  | cagcctggattttcctcact |
| IL11 | gctgagagacaaattcccag |
|  | ggtaggaaaacaggtctgct |
| CXCL2 | agcagacacagaccctccga |
|  | agcggccactagaagcagga |
| CXCL8 | AGCCCGTGTCAACATGACTTCC |
|  | TTGTGTTGGCATCTTTACTGA |
| CCL2 | CTCCCACACCGAAGCTTGAA |
|  | TAATTGCATCTGGCTGGGCA |
| SERPINB2 | tcatccccatgtctgaactt |
|  | gtgtttgccacgtaaagttc |
| TFPI | aggacagatttttggggctt |
|  | attggtggcaactttgcatc |
| THBS1 | gtttccacacaggctctctg |
|  | gccacacacatgcaacagaa |
| THBD | atgctccgcgttctgctcctt |
|  | agcagtcatgctcgacgcact |
| Occludin | ttatgcacccagcaacgac |
|  | ctggctgagaaagcattggt |
| E-selectin | CTGAGCACCTGCAATGTACC |
|  | GCTTTACACGTTGGCTTCTTG |
| VCAM-1 | CATTCCATGGTGTCCCAGA |
|  | TCCAAACTCTTCGTTTCCTTG |
| ICAM-1 | GAGGAGCTGTTCAGGCAGTC |
|  | CATCCGGAACGTGACATTG |
| IL6 | GCTGCTTCTGGTGATGGCTACTGCC |
|  | TGAAACTCCACAAGACCGGTGGTGA |
| Rpl13a | attgtggccaagcaggtact |
|  | cagaaatgttgatgccctcac |

**Supplementary Table 1: Primer sequences of porcine genes for real-time PCR**

**Supplementary Figure 1**

**
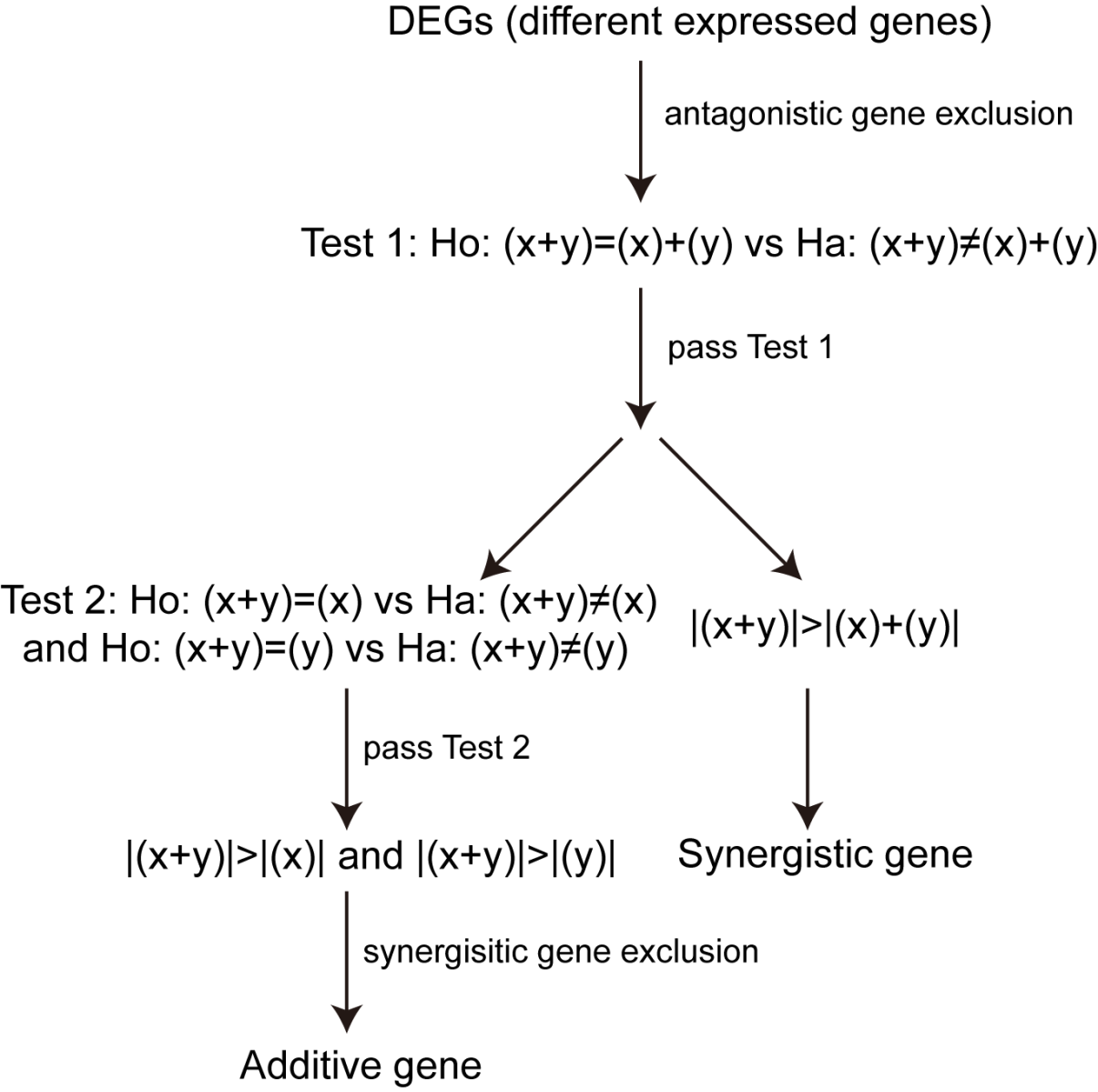
**

**Supplementary Figure 1: The flow chart of additive and synergistic genes definition.**

**Supplementary Figure 2**


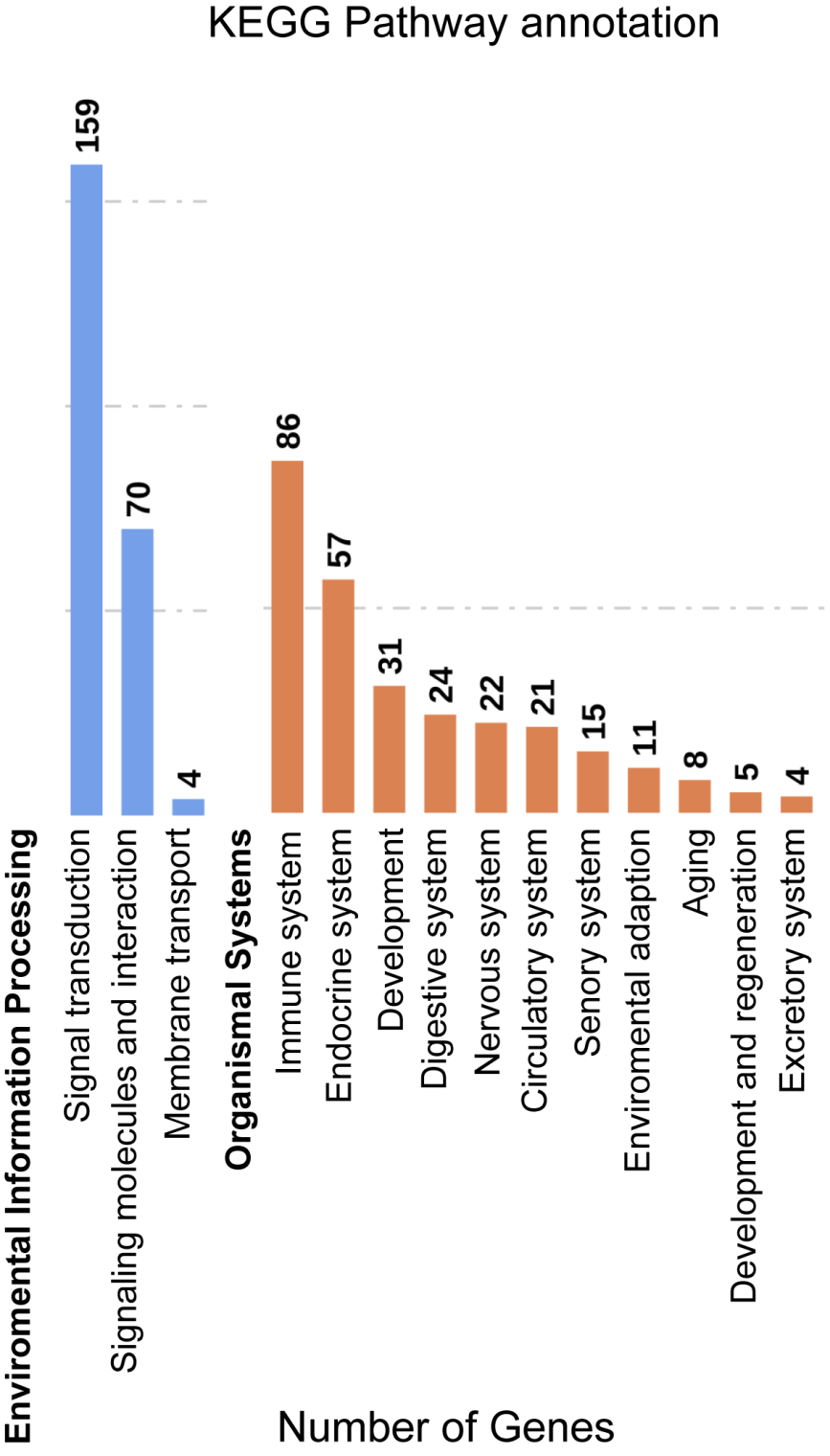


**Supplementary Figure 2: Immune system represented as the top enrichment containing 86 genes.**

KEGG pathway annotation of ASGs between control group and IL-17 plus TNF-α group.

**Supplementary Figure 3**


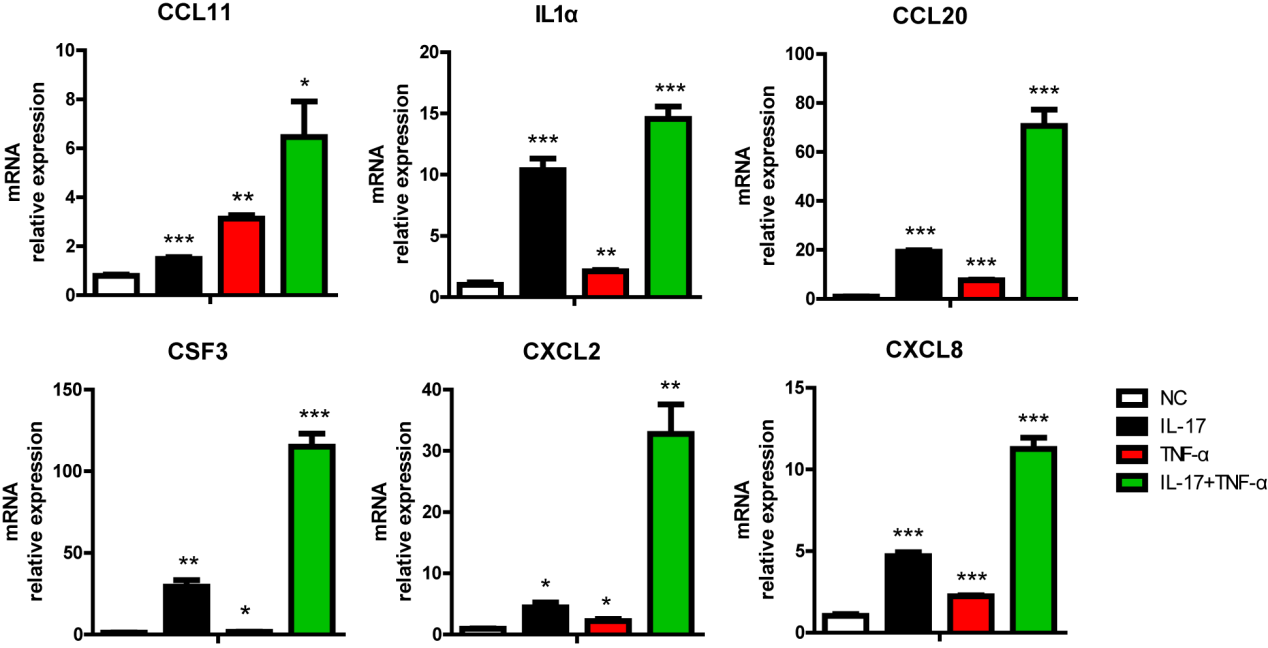


**Supplementary Figure 3: IL-17 and TNF-α additively or synergistically induced chemokine or cytokine expression in *GGTA1/CMAH* double-knockout (DKO) PAECs.**

*GGTA1/CMAH* DKO PAECs were treated with rhIL-17 (100 ng/mL), rhTNF-α (2 ng/mL), or rhIL-17 plus rhTNF-α for 0, or 6 h. Induction of CCL11, IL1α, CCL20, CSF3, CXCL2 or CXCL8 mRNA was measured by real-time PCR.

Data are representative of at least three independent experiments (mean±SEM). **p*<0.05, ***p*<0.01, ****p*<0.001 by Student’s t test

**Supplementary Figure 4**


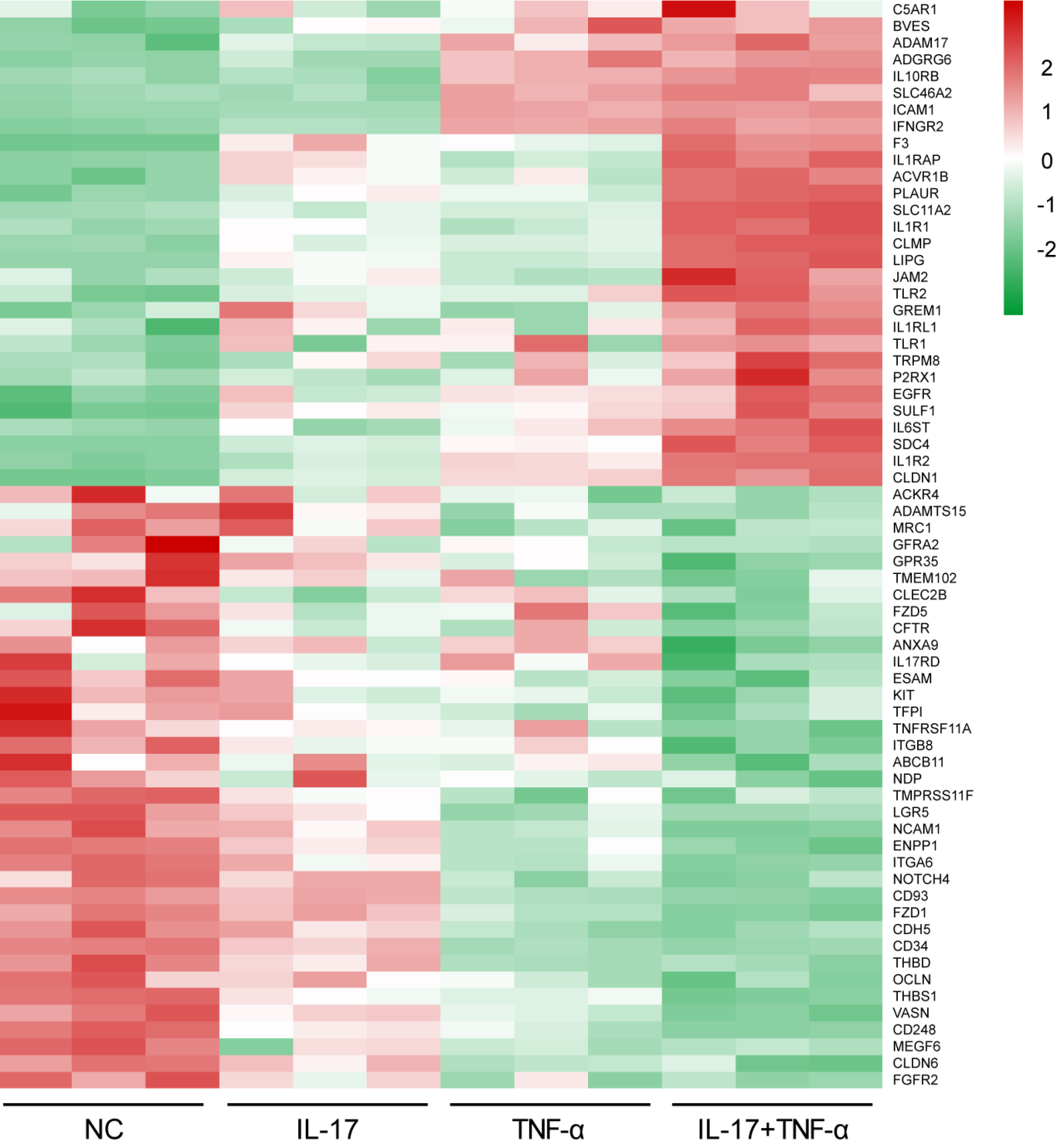


**Supplementary Figure 4: IL-17 and TNF-α addictively or synergistically regulated 65 cell surface genes expression.**

Heatmap showing cell surface proteins of ASGs in control group, IL-17 group, TNF-α group, or IL-17 plus TNF-α group.

**Supplementary Figure 5**


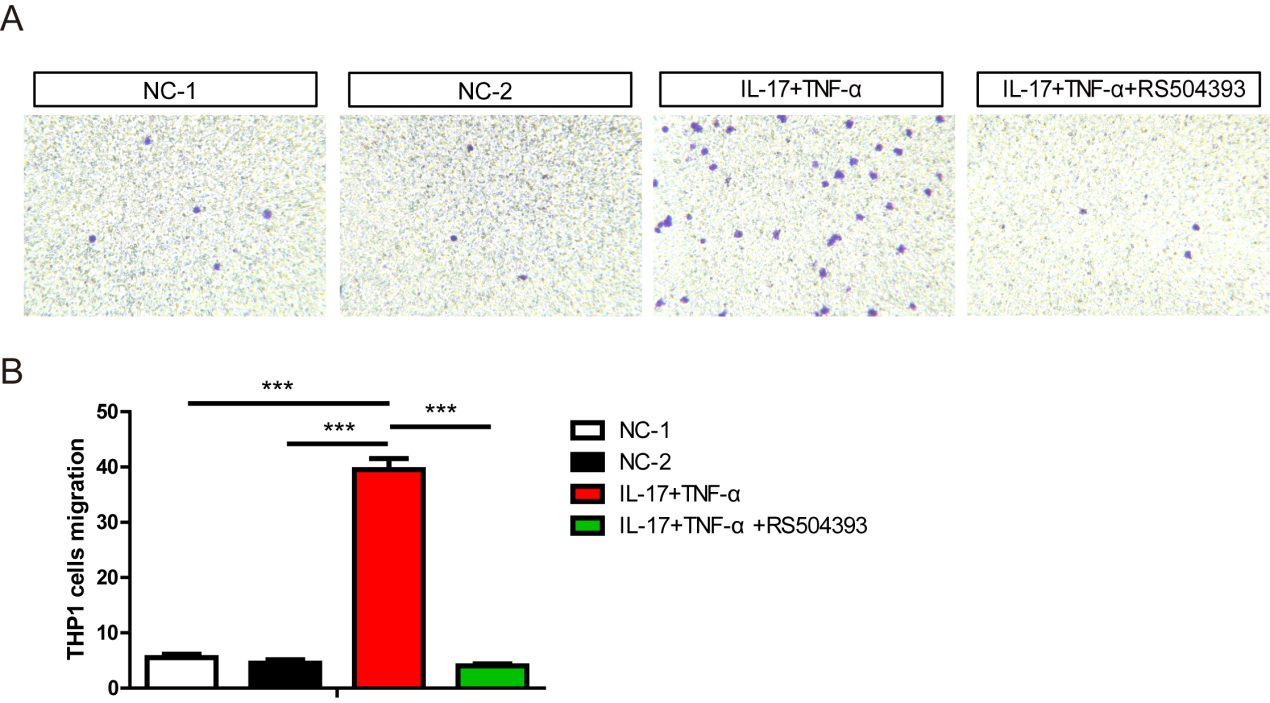


**Supplementary Figure 5: The combination of IL-17 with TNF-α increased the chemotaxis of THP-1 cells through inducing CCL2 production.**

(A) PAECs were treated by with IL-17 plus TNF-α for 48 h, the supernatant was collected for chemotaxis assay, the supernatant from PAECs without IL-17 or TNF-α treatment as negative control-1 (NC-1), the supernatant from PAECs without IL-17 or TNF-α treatment was added IL-17 plus TNF-α as negative control-2 (NC-2). For IL-17+TNF-α+RS504393 group, THP-1 cells were treated with 10μM CCR2 specific inhibitor RS504393 for 1h.

(B) The number of migrating cells per field was determined as in (A).

**Supplementary Figure 6**

**Supplementary Figure 6: IL-17 or TNF-α didn’t affect the viability of PAECs.**

PAECs were treated with or without hIL-17 (100 ng/mL), hTNF-α (2 ng/mL), or hIL-17 plus hTNF-α for 48 h and assess the viability of PAECs with CCK8. The absorbance values of wells were measured with OD 450.
